# Theoretical Research on the Axial Compression Capacity of Reinforced Concrete Mid-Long Columns Reinforced with Ultra-High-Performance Concrete

**DOI:** 10.3390/ma18020300

**Published:** 2025-01-10

**Authors:** Xiaolong Tong, Zhengwu Liao, Wei Liu, Huajing Guo, Jianliang Wu

**Affiliations:** 1 School of Civil and Architectural Engineering, Hunan Institute of Science and Technology, Yueyang 414000, China; 2 Guangzhou Municipal Engineering Testing Co., Ltd., Guangzhou 510520, China

**Keywords:** ultra-high-performance concrete (UHPC), UHPC hardening, RC medium–long column, axial compression capacity, tangent modulus theory

## Abstract

Ultra-high-performance concrete (UHPC) is a cement-based composite material characterised by exceptional strength, low porosity and high durability, making it highly promising for reinforcement engineering. Based on the theory of tangential modulus, a calculation method has been developed for the axial compression capacity of reinforced concrete (RC) medium and long columns strengthened with UHPC, using the constitutive relation of materials and internal and external force balance conditions. This study analysed the influence of UHPC reinforcement layer thickness, reinforced layer, reinforcement ratio, column slenderness ratio and initial load level of core columns on the bearing capacity of reinforced columns. The results indicated that the bearing capacity of the mid-long columns increases with the thickness of the UHPC reinforcement layer and its reinforcement ratio. In addition, the bearing capacity decreases with the increase in the column slenderness ratio and initial load level of the core column.

## 1. Introduction

Ultra-high-performance concrete (UHPC), a fibre-reinforced cement-based composite material, exhibits exceptional mechanical properties and durability. Its ultra-high strength, low porosity and high durability make it highly suitable for reinforcing existing concrete structures. In recent years, many buildings have experienced structural damage owing to factors such as construction quality, approaching the end of their design life and improper use, affecting their safety and service life. UHPC is an innovative concrete type designed using the maximum density theory. Compared to conventional reinforced concrete, UHPC offers significant advantages. Its ultra-high strength reduces the amount of concrete needed for the same load-bearing capacity, which significantly decreases the weight of reinforced columns and reduces foundation stress. Additionally, UHPC’s dense microstructure provides superior impermeability and durability. These attributes position UHPC to play an increasingly critical role in reinforcing and refurbishing existing concrete structures [1,2,3].

Common reinforcement methods include the ordinary reinforced concrete section enlargement method, FRP method and steel plate bonding method [4,5,6]. The adhesive steel plates or fibre cloth can seal cracks and improve section strength, while they struggle to address the general decline in the stiffness of old columns. Conversely, using ordinary concrete to increase the pier column’s section can effectively enhance its strength and stiffness, though it has limited impact on bearing capacity improvement. The increased section method is highly applicable, with minimal technical difficulty in construction and good economic benefits, making it widely suitable for reinforcing reinforced concrete columns. Research on strengthening RC columns through the increased cross-section method is of great practical significance.

RC axial compression columns can be classified into three categories based on their slenderness ratio (*l*_0_/*b*): short columns (*l*_0_/*b* ≤ 8), medium columns (8 < *l*_0_/*b* ≤ 30), and slender columns (*l*_0_/*b* > 30). The failure modes of these columns correspond to material failure, elastoplastic failure, and elastic failure, respectively. In practical engineering, medium and long columns are more commonly used. The failure modes of long columns differ from those of short columns, requiring in-depth study of medium and long columns [7,8,9].

Thus far, there have been many studies concentrated on the bearing capacity of reinforced RC columns. Farzad et al. [10] studied structural performance and durability enhancement of circular reinforced concrete columns repaired with UHPC. Bolina et al. [11] experimentally analysed the thermal properties of a number of UHPCs and studied the thermal fields of the cross-sections of conventional UHPC beams using finite element method. Bolina et al. [12] investigated the thermal field of NSC and equivalent UHPC cross-sections of typical frame structural elements exposed to high temperatures. Nettis et al. [13] studied the fragility of existing PC girder bridges considering traffic loads, accounting for corrosion-induced effects. Hung et al. [14] carried out tests to investigate the compressive behaviour of UHPC-reinforced short columns. Wang et al. [15] studied the mechanical and fatigue properties of UHPC-reinforced NC composite beams under recirculation load. Khodayari et al. [16] used UHPC to repair damaged reinforced concrete T-beams and studied the mechanical properties and bearing capacity of the repaired T-beams under cyclic and ultimate bending loads. Zhao et al. [17] used a direct shear test to study the shear properties of the UHPC-NC interface. Zhang et al. [18] studied the performance of RC columns strengthened with a thin UHPC jacket under cyclic loads.

The bearing capacity of axial compression members represents the upper limit of the load they can bear, serving as a crucial parameter for strength assessment and preliminary section estimation in engineering design [19]. Therefore, the analysis on the UHPC-reinforced axial compression capacity of RC mid-long columns has significant theoretical importance. However, the current theoretical studies on the bearing capacity of reinforced RC columns by domestic and international scholars predominantly address short columns, with limited research on medium and long columns.

In this study, a calculation method for the axial compression capacity of RC axial columns reinforced with UHPC was derived based on the tangent modulus theory. Furthermore, the impact of UHPC-reinforced layer thickness, reinforcement ratio, column slenderness ratio and initial load level of the core column on the bearing capacity of the reinforced column was studied using this method.

## 2. Calculation Method

The classical Euler formula is limited to the elastic instability of slender rods. However, the stability problem of RC axial compression medium–long columns involves the elastic–plastic stability of moderately flexible compression rods. In such cases, the material stress during concrete failure exceeds the elastic proportional limit, rendering the classical Euler formula inapplicable. The tangent modulus theory addresses this by replacing the material’s elastic modulus *E* in the classical Euler formula *N_c r_* = *π*^2^*EI/l*_0_^2^ with the tangent modulus *E_t_* = *dσ/dε*. This modification allows the Euler formula to be used to determine the elastoplastic stability capacity of the compression rod. Shanley [20,21] validated the tangent modulus theory and demonstrated that the ultimate load derived from the tangent modulus represents the lower limit of the elastic–plastic ultimate load.

**Figure 1:**
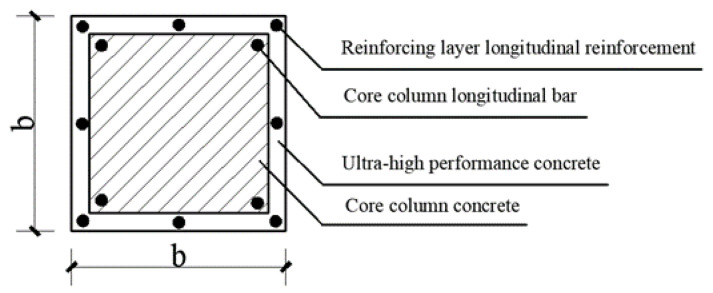
Schematic diagram of the composite cross-section.

The UHPC-reinforced RC column consists of two types of materials: the core column and the external UHPC reinforcement layer. The cross-section of the reinforced column includes both these components. A diagram illustrating the cross-section of the reinforced column is shown in Figure 1.

Based on the principle of rigidity superposition, the stiffness of the reinforced column is the sum of the stiffness of the core column concrete and rebar and the UHPC reinforcement layer and rebar. The elastic–plastic stability bearing capacity Nu of the reinforced column can be expressed as follows:

(1) $$N_{u}=\frac{\pi^{2}}{l_{0}^{2}}\left(E_{c1}^{t}I_{c1}+E_{s1}^{t}I_{s1}+E_{c2}^{t}I_{c2}+E_{s2}^{t}I_{s2}\right)$$

where *l*_0_ is the calculated length of the reinforced column, $E_{c1}^{t}$ is the tangential modulus of core column concrete, *I_c_*_1_ is the moment of inertia of the concrete section of the core column against the mandrel, $E_{s1}^{t}$ is the tangent modulus of core column reinforcement, *I_s_*_1_ is the moment of inertia of the rebar of the core column against the section mandrel calculated by multiplying the area of each side of the rebar by the square of the distance between the rebar and the section geometry,$\;E_{c2}^{t}$ is the tangential modulus of reinforced layer UHPC, *I_c_*_2_ is the moment of inertia of the reinforced concrete section against the mandrel,$\;E_{s2}^{t}$ is the tangential modulus of reinforcement layer, *I_s_*_2_ is the moment of inertia of the reinforcement layer against the cross-section mandrel calculated using the same method as *I_s_*_1_.

The research in [22] demonstrated that the strain in the section will not exceed the peak strain of the reinforced concrete axial compression member when elastoplastic failure occurs. In addition, the tangential modulus theory in this paper only considers the compression constitutive of concrete. Therefore, the cracking of concrete did not occur in this study, and the influence of cracking of concrete was not assumed in the equations.

### 2.1. Reinforcement Constitutive Relation

The tangent modulus $E_{s}^{t}$ of the steel bar in the elastoplastic stage can be represented as a quadratic parabola [23]:

(2) $$E_{s}^{t}=\frac{d\sigma_{s}}{d\varepsilon_{s}}=\frac{\left(f_{y}-\sigma_{s}\right)\sigma_{s}}{\left(f_{y}-f_{p}\right)f_{p}}E_{s}\;$$

where *f_y_* is the yield strength of reinforcement; *f_p_* is the proportional limit of the steel bar and is 0.8 times the yield strength [24]; and *E_s_* is the elastic modulus of the steel bar.

By transforming Formula (2), the following can be obtained:

(3) $$\frac{d\sigma_{s}}{\left(f_{y}-\sigma_{s}\right)\sigma_{s}}=\frac{E_{s}d\varepsilon_{s}}{\left(f_{y}-f_{p}\right)f_{p}}$$

Further refinement of the abovementioned formula yields the following:

(4) $$\frac{d\sigma_{s}}{\sigma_{s}}+\frac{d\sigma_{s}}{\left(f_{y}-\sigma_{s}\right)}=\frac{{f_{y}E}_{s}d\varepsilon_{s}}{\left(f_{y}-f_{p}\right)f_{p}}$$

By integrating both sides of Formula (4), the following can be obtained:

(5) $$\ln\left(\frac{\sigma_{s}}{f_{y}-\sigma_{s}}\right)=\frac{{f_{y}E}_{s}\varepsilon_{s}}{\left(f_{y}-f_{p}\right)f_{p}}+C$$

In the abovementioned formula, *C* is an integral constant. Under the conditions $\varepsilon_{s}=\varepsilon_{p}$ and $\sigma_{s}=f_{p}$, one can obtain $C=\frac{f_{y}}{f_{y}-f_{p}}-ln\left(\frac{f_{y}}{f_{y}-f_{p}}\right)$.

By taking the reciprocals on both sides of Formula (5), the following can be obtained:

(6) σs=fy1+k1−ke1−εsεp1−k 

where *ε_p_* is the strain corresponding to the proportional limit of the steel bar, and the ratio *k* of the proportional limit of steel bar *f_p_* to the yield strength *f_y_* is preferred to be 0.8 [24].

By substituting *k* = 0.8 into Formula (6), the stress–strain relationship and the tangential modulus of the steel bar for elastoplastic analysis can be determined:

(7) σs=Esεs          Est=Es                  εs≤εpσs=4fy4+e51−εsεp  Est=fy−σsσsfy−fpfpEs   εs>εp 

According to the constitutive relation and tangential modulus expression derived from the abovementioned theory, if the material strain at a certain time is known, the stress and tangential modulus of the material in this strain state can be calculated.

### 2.2. Constitutive Relation of the Concrete

The research [22] demonstrated that the strain in the section could not exceed the peak strain ε_0_ of the concrete when elastoplastic instability failure occurs in the reinforced concrete axial compression member. In this study, analysis on the constitutive relation of ordinary concrete was performed based on the Code for Design of Concrete Structures [25] and the constitutive relation of UHPC according to the Technical Specification for Reactive Powder Concrete Structures [26]. Note that different stress–strain relationships and tangential moduli are employed for ordinary concrete and UHPC. The non-linearity of the concrete can be taken into account in the following equations.

The stress–strain relationship of ordinary concrete under pressure is as follows:

(8) $$\sigma_{c}=f_{c}\left[1-{\left(1-\frac{\varepsilon_{c}}{\varepsilon_{0}}\right)}^{2}\right]\;\;\;\;\;\varepsilon_{c}\leq \varepsilon_{0}$$

By deriving Formula (8), the tangent modulus of ordinary concrete can be obtained:

(9) $$E_{c}^{t}=\frac{d\sigma_{c}}{d\varepsilon_{c}}=2f_{c}\left(\frac{1}{\varepsilon_{0}}-\frac{\varepsilon_{c}}{\varepsilon_{0}^{2}}\right)\;\;\;\varepsilon_{c}\leq \varepsilon_{0}$$

The stress–strain relationship of UHPC compression is as follows:

(10) $$\sigma_{c}=f_{c}\left[1-{\left(1-\frac{\varepsilon_{c}}{\varepsilon_{0}}\right)}^{n}\right]\;\;\;\varepsilon_{c}\leq \varepsilon_{0}$$

(11) $$n=1.2-0.001\left(f_{cu,k}-100\right)\;\;\;\varepsilon_{c}\leq \varepsilon_{0}$$

By deriving Formula (10), the tangent modulus of UHPC can be obtained:

(12) $$E_{c}^{t}=\frac{d\sigma_{c}}{d\varepsilon_{c}}=nf_{c}\left(\frac{1}{\varepsilon_{0}}-\frac{\varepsilon_{c}}{\varepsilon_{0}^{2}}\right)\;\;\;\;\varepsilon_{c}\leq \varepsilon_{0}\;$$

### 2.3. Formula Derivation

Based on the bearing capacity of ordinary reinforced concrete in Mao’s research [27], once elastic–plastic instability occurs in a reinforced RC axial compression medium–long column, the ultimate bearing capacity can be calculated using tangential modulus theory:

(13) $$N_{u}=\frac{\pi^{2}}{l_{0}^{2}}\left(E_{c1}^{t}I_{c1}+E_{s1}^{t}I_{s1}+E_{c2}^{t}I_{c2}+E_{s2}^{t}I_{s2}\right)$$

The equivalent reduced stiffness of the combined section after reinforcement is defined as follows:

(14) $${E_{sc}^{t}I_{sc}=E}_{c1}^{t}I_{c1}+E_{s1}^{t}I_{s1}+E_{c2}^{t}I_{c2}+E_{s2}^{t}I_{s2}$$

where $E_{sc}^{t}$ is the equivalent tangent modulus of composite cross-section material; *I_sc_* is the equivalent moment of inertia of the combined section, which is the total moment of inertia of the section.

Then, Formula (13) can be re-expressed as follows:

(15) $$N_{u}=\frac{\pi^{2}E_{sc}^{t}I_{sc}}{l_{0}^{2}}$$

$E_{sc}^{t}$ can be calculated as follows [27]:

(16) $$E_{sc}^{t}=\frac{E_{c1}^{t}A_{c1}+E_{s1}^{t}A_{s1}^{\prime }+E_{c2}^{t}A_{c2}+E_{s2}^{t}A_{s2}^{\prime }}{A_{sc}}\;$$

where *A_sc_* is the equivalent area of the section,$\;A_{sc}=A_{c1}+A_{s1}^{\prime }+A_{c2}+A_{s2}^{\prime }=b^{2}$, *A_c_*_1_ is the core column concrete area,$\;A_{s1}^{\prime }$ is the core column reinforcement area, *A_c_*_2_ is the reinforced layer concrete area, and$\;A_{s2}^{\prime }$ is the reinforcing layer reinforcement area.

According to the principle that the total moment of inertia of the section is equal to the sum of the moments of inertia of each component,

(17) $$I_{sc}=I_{c1}+I_{s1}+I_{c2}+I_{s1}=\frac{b^{4}}{12}\;$$

In the formula, *b* is the side length of the combined section after reinforcement.

The core column is subjected to long-term load which is assumed to be *N*_1_, and the concrete strain *ε_c_*_1,1_ under long-term load can be obtained. The geometric characteristic value of the combined section of the reinforced member can be calculated by Equations (16) and (17).

Then, the equivalent critical stress *σ_sc_* of the combined cross-section in the critical state is as follows:

(18) $$\sigma_{sc}=\frac{N_{u}}{A_{sc}}=\frac{\pi^{2}E_{sc}^{t}I_{sc}}{l_{0}^{2}A_{sc}}$$

The equivalent slenderness ratio is defined as follows:

(19) $$\lambda_{sc}=\frac{l_{0}}{i_{sc}}=\frac{l_{0}}{\sqrt{I_{sc}/A_{sc}}}=\sqrt{12}\frac{l_{0}}{b}$$

By substituting Formula (19) into Formula (18), Formula (18) becomes

(20) $$\sigma_{sc}=\frac{\pi^{2}E_{sc}^{t}}{\lambda_{sc}}$$

The equivalent compressive strength *f_sc_* of the cross-sectional material is defined as follows:

(21) $$f_{sc}=\frac{f_{c1}A_{c1}+f_{s1}^{\prime }A_{s1}^{\prime }+{f_{c2}A}_{c2}+{f_{s2}^{\prime }A}_{s2}^{\prime }}{A_{sc}}$$

where *f_c_*_1_ is the design value of compressive strength of core column concrete;$\;f_{s1}^{\prime }$ is the design value of compressive strength of core column reinforcement; *f_c_*_2_ is the design value of compressive strength of reinforced concrete; and$\;f_{s2}^{\prime }$ is the design value of compressive strength of reinforcement layer.

When the critical force equals the material failure-bearing capacity, the component failure is determined by the material’s strength, and stability checks are not required. In this case,

(22) $$\sigma_{sc}A_{sc}=f_{c1}A_{c1}+f_{s1}^{\prime }A_{s1}^{\prime }+{f_{c2}A}_{c2}+{f_{s2}^{\prime }A}_{s2}^{\prime }=f_{sc}A_{sc}$$

As a result, the equivalent compressive strength *f_sc_* can be rewritten as follows:

(23) $$\frac{\pi^{2}E_{sc,0}^{t}}{\lambda_{sc,0}}=f_{sc}$$

where $E_{sc,0}^{t}$ is the equivalent tangential modulus of composite section material when the critical force is equal to the strength bearing capacity.

It can be observed that the limit equivalent slenderness ratio *λ_sc,_*_0_ for material failure and elastoplastic failure of the reinforced column is as follows:

(24) $$\lambda_{sc,0}=\pi \sqrt{\frac{E_{sc,0}^{t}}{f_{sc}}}$$

When *λ_sc_* ≤ *λ_sc,_*_0_, the bearing capacity of the reinforced column is controlled by the strength of the material. Elastic–plastic failure occurs when *λ_sc_* > *λ_sc,_*_0_.

When *λ_sc_* > *λ_sc,_*_0_, the ultimate bearing capacity of the reinforced column can be determined according to the tangent modulus theory. Before reinforcement, under the action of load $N_{1}$ in the first stage, the stress $\sigma_{c1,1}$ of the core column concrete is as follows:

(25) $$\sigma_{c1,1}=\frac{N_{1}}{A_{c1}+{nA}_{s1}^{\prime }}$$

where n is the ratio of elastic modulus of reinforced concrete to the core column.

From the stress *σ_c_*_1,1_ of the core column concrete under one-stage load, the strain *ε_c_*_1,1_ of the core column concrete under one-stage load can be inversely calculated using the constitutive relation of concrete, specifically Formula (2), as follows:

(26) $$\varepsilon_{c1,1}=\left(1-\sqrt{1-\frac{\sigma_{c1,1}}{f_{c1}}}\right)\varepsilon_{0}\;$$

According to the assumption of a plane section, the longitudinal deformation of the component is coordinated. The strain *ε_s_*_1,1_ = *ε_c_*_1,1_ of the core column reinforcement under the action of *N*_1_.

When elastic–plastic failure occurs, assuming the member is an ideal straight rod, it remains straight up to the moment before buckling. At this point, under an external load *N_u_*, the concrete of the reinforced layer has a longitudinal strain *ε_c_*_2_. Owing to deformation coordination, the strain *ε_s_*_2_ = *ε_c_*_2_ of the reinforced layer. The strain of concrete and reinforcement of the core column is *ε_c_*_1_ = *ε_s_*_2_ = *ε_c_*_1,1_ + *ε_c_*_2_, and the tangent modulus of each part can be calculated according to the strain. Under this longitudinal strain state, the stresses of each part, $\sigma_{c1}$, $\sigma_{s1}^{\prime }$, $\sigma_{c2}$, $\sigma_{s2}^{\prime }$, can be calculated by their constitutive relations. The ultimate bearing capacity $N_{u}$ of the reinforced column can be expressed as follows:

(27) $$N_{u}=\sigma_{c1}A_{c1}+\sigma_{s1}^{\prime }A_{s1}^{\prime }+\sigma_{c2}A_{c2}+\sigma_{s2}^{\prime }A_{s2}^{\prime }$$

where $\sigma_{c1}$ is the stress of core column concrete when the pillar fails;$\;\sigma_{s1}^{\prime }$ is the stress of the core column reinforcement when the column is broken;$\;\sigma_{c2}$ is the stress of the reinforced concrete when the pillar is broken;$\;\sigma_{s2}^{\prime }$ is the stress of the reinforcement layer when the pillar fails.

Under the limit state of carrying capacity, Formulas (18) and (27) are equal, that is,

(28) $$\frac{\pi^{2}E_{sc}^{t}A_{sc}}{\lambda_{sc}^{2}}=\sigma_{c1}A_{c1}+\sigma_{s1}^{\prime }A_{s1}^{\prime }+\sigma_{c2}A_{c2}+\sigma_{s2}^{\prime }A_{s2}^{\prime }\;$$

### 2.4. Calculation Procedure

From the abovementioned derivation and analysis, it is evident that each variable in Formula (28) represents the longitudinal strain and is a function of the concrete strain $\varepsilon_{c2}$ of the reinforced layer. Therefore, $\varepsilon_{c2}$ in the critical state can be determined using Formula (28), which in turn allows for determining the column bearing capacity $N_{u}$. This process must be completed iteratively, and the specific steps are as follows:

(1) Calculate $\varepsilon_{c1,1}$ of the core column under the one-stage load $N_{1}$.
(2) Calculate the geometric properties of the combined section of the reinforced member, including $A_{c1}$, $A_{s1}^{\prime }$, $A_{c2}$, $A_{s2}^{\prime }$, $A_{sc}$, $I_{c1}$, $I_{s1}$, $I_{c2}$, $I_{s2}$ and $I_{sc}$.
(3) Set $\varepsilon_{c2}$ for the reinforcement layer part in which $\sigma_{c1}$, $\sigma_{s1}^{\prime }$, $E_{c1}^{t}$ and $E_{s1}^{t}$ can be determined based on the constitutive relationship between the concrete and the steel bar. For the core column, $\varepsilon_{c2}$ and $\varepsilon_{c1,1}$ are superimposed, and the corresponding $\sigma_{c1}$, $\sigma_{s1}^{\prime }$, $E_{c1}^{t}$ and $E_{s1}^{t}$ are determined by the constitutive relationship between the concrete and the steel bar of the core column.
(4) Substitute the parameters into Equation (28) and check if the left and right sides of the equation are equal.
(5) The value is the critical load if the left side equals the right side. If they are not equal, increase $\varepsilon_{c2}$ if the left side is greater than the right side, and decrease $\varepsilon_{c2}$ if the left side is less. Repeat steps (3)–(4) until the left and right sides are equal, thus obtaining the critical load.

## 3. Verification of the Calculation Results

Research on the axial compression performance of RC medium and long columns reinforced with UHPC is scarce, with no reported or relevant experimental studies. Base on the tests in Mao’s study [28], the basic parameters for specimen design, used as a reference in this paper, are shown in Table 1. The grade strength of concrete for the core columns is C20, and for the reinforced layers, it is C30. The theoretical carrying capacities of ZY-1, ZY-2 and ZY-3 were calculated using the derived theoretical method. The developed calculation method was verified by using the constitutive of ordinary concrete, and the relative difference between calculation and test was acceptable, shown as Table 2. Therefore, the equations can be treated as validated, and the results are reliable. It also can be obtained that the theoretical calculation results in this paper are slightly higher than those in the literature [28] and are closer to the measured carrying capacity values.

## 4. Case Study

Research on the axial compression performance of RC medium and long columns reinforced with UHPC is scarce, with no reported or relevant experimental studies. Based on the tests in Mao’s study [28], this paper refers to the test and material parameters in reference [28] as examples for calculation.

**Figure 2:**
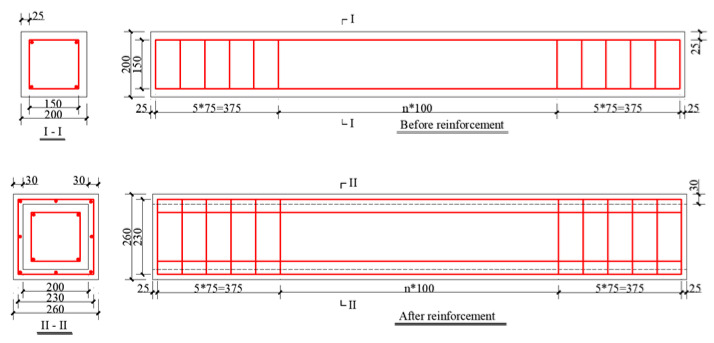
Section size diagram of concrete-reinforced column.

The rebar was HRB335, and the core columns were made of C20 ordinary concrete. The diameter of the longitudinal reinforcement was 12 mm, and the diameter of the stirrup was 6 mm. The section size of the core column was 200 mm × 200 mm. The thickness of the reinforcement layer was 30 mm, and UHPC was used for reinforcement. The designed parameters of the specimen are shown in Table 3, the mechanical properties of the material are shown in Table 4 and Table 5, and the cross-section size of the reinforced column is shown in Figure 2.

Note that the above values are substituted into the calculation iterative equations using the iterative approach described in Section 2.4. As a result, the axial compression capacity of the specimen JGZ-1 can be obtained as 1935.7 KN, namely C20 ordinary concrete column strengthened with 30 mm thick UHPC, and the axial compression capacity of the specimen JGZ-2 can be obtained as 3307 KN where the thickness of the reinforced layer is 50 mm, as shown in Table 6.

The results illustrate that UHPC for reinforcement enables reducing the thickness of the reinforcement layer, compared with that of the ordinary-concrete-reinforced column, which can significantly reduce the amount of concrete and the structure’s weight. UHPC is a new type of concrete configured according to the maximum compactness theory, and its specific compressive strength is about 7 times that of ordinary concrete and 2 times that of ordinary steel. In addition, the creep of UHPC under long-term load is very small, about 1/10 of ordinary concrete [29]. UHPC is suitable for the reinforcement and reconstruction of concrete structures.

Therefore, theoretical research on the axial compression capacity of RC mid-long columns reinforced with UHPC has been performed, and the conclusions based on this study have great significance.

## 5. Parameter Analysis

The variables selected for analysis were the thickness of the UHPC reinforcement layer, slenderness ratio, reinforcement layer configuration and initial pressure level. RPC120 was used for reinforcement, and 8B8 was adopted for longitudinal reinforcement of the reinforcement layer.

The parameter values were as follows. The thickness of the UHPC reinforcement layer: 30 mm, 40 mm, 50 mm and 60 mm; Slenderness ratio: 3.8, 6.9, 7.7, 9.2, 11.5, 15.4, 19.2 and 23.1; Reinforcement layer configuration: 0, 8B6, 8B8, 8B10 and 8B12; Initial pressure levels: 0, 0.2, 0.4 and 0.6.

### 5.1. Impact of Hardening Layer Thickness

**Figure 3:**
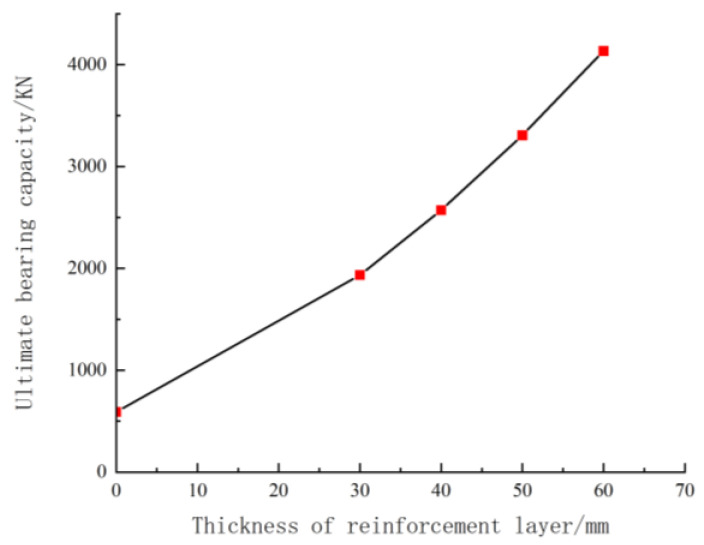
Effect of reinforcement layer thickness on bearing capacity.

As shown in Figure 3, the bearing capacity increases significantly with the thickness of the reinforced layer. This occurs because the material used in the reinforced layer is UHPC, which has a much higher compressive strength than ordinary concrete. Consequently, the tangent modulus value is higher, leading to a greater value of $E_{sc}^{t}I_{sc}$. When the thickness of the reinforced layer is 30 mm, the bearing capacity is approximately 3.7 times that of the unreinforced column. When the thickness increases to 40 mm, the bearing capacity is approximately 1.28 times that of the 30 mm reinforced layer. Each additional 10 mm increase in thickness results in a bearing capacity that is approximately 1.2 times the previous thickness.

### 5.2. Impact of Slenderness Ratio

**Figure 4:**
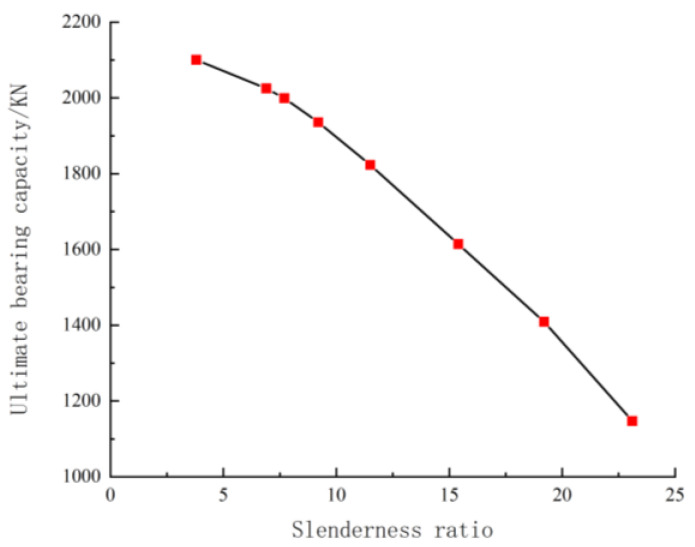
Effect of slenderness ratio on bearing capacity.

As shown in Figure 4, the bearing capacity decreases with an increasing slenderness ratio. However, the relationship is not linear. When the slenderness ratio exceeds 11.5, the bearing capacity decreases significantly. The bearing capacity at$\;l_{0}/b$ = 9.2 is approximately 97% of that at $l_{0}/b$ = 3.8, and the bearing capacity at$\;l_{0}/b$ = 23.1 is approximately 76% of that at$\;l_{0}/b$ = 3.8.

### 5.3. Influence of Reinforcement Layer

**Figure 5:**
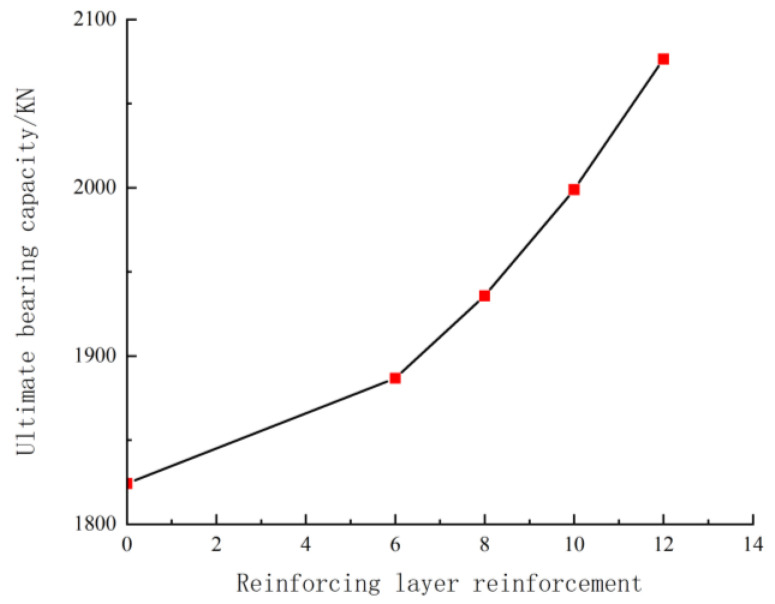
Effect of reinforcement on bearing capacity.

As shown in Figure 5, the bearing capacity increases with the amount of reinforcement in the reinforced layer. This occurs because the tangential modulus of steel bars is much higher than that of concrete. As the number of steel bars increases, their contribution to the overall stiffness becomes more significant, leading to an increase in the value of $E_{sc}^{t}I_{sc}$ and, consequently, an increase in the bearing capacity.

### 5.4. Influence of the Initial Pressure Level

**Figure 6:**
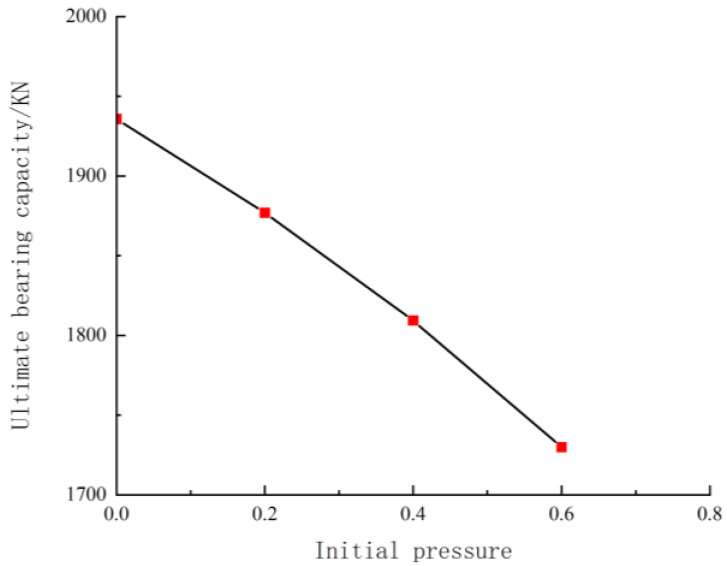
Effect of initial pressure level on bearing capacity.

As shown in Figure 6, the bearing capacity gradually decreases with an increase in the initial pressure level. The higher the initial load, the lower the total bearing capacity. This occurs because the initial strain in the original column of the secondary stressed component prevents ordinary concrete and UHPC from reaching their peak compressive strain simultaneously under the limit state. After the core column concrete reaches its peak load, the component can continue to load, further decreasing the strength utilisation rate of the core column concrete, resulting in a lower total bearing capacity.

## 6. Conclusions

(1) Based on the tangent modulus theory, a calculation method was derived for the axial compression capacity of RC medium and long columns strengthened with UHPC. A comparison with the experiments cited in references demonstrates that the theoretical calculation values closely align with the experimental results.
(2) Compared with that of the ordinary concrete-reinforced column, the bearing capacity of the UHPC-reinforced column can be greatly enhanced, and UHPC for reinforcement enables reduction in the thickness of the reinforcement layer.
(3) The bearing capacity of the reinforced column increases significantly with the thickness of the reinforced layer. The bearing capacity decreases with increase in the slenderness ratio. When >1.5, the bearing capacity of the reinforced column decreases significantly. Additionally, the bearing capacity of the reinforced column increases with the amount of reinforcement in the reinforced layer. Conversely, the bearing capacity decreases with an increase in the initial pressure level.

## Figures and Tables

**Table 1:** Design parameters of specimens.

Specimen Number	Core Column Section Dimensions/mm	Section Dimensions of the Reinforced Rear Column/mm	Specimen Length/mm	Core Column Main Bar	Reinforcing Layer Main Reinforcement
ZY-1	200 × 200	300 × 300	1800	4B12	8B12
ZY-2	200 × 200	300 × 300	2400	4B12	8B12
ZY-3	200 × 200	300 × 300	3000	4B12	8B12

**Table 2:** The bearing capacity values.

Specimen Number	Calculated Value/KN	Measured Value [28]/KN	Relative Difference
ZY-1	1730	1900	9.83%
ZY-2	1722	1936	12.43%
ZY-3	1712	1810	5.72%

**Table 3:** Designed parameters of specimens.

Specimen Number	Core Column Section Dimensions/mm	Section Dimensions of the Reinforced Rear Column/mm	Specimen Length/mm	Core Column Main Bar	Reinforcing Layer Main Reinforcement
JGZ-1	200 × 200	260 × 260	2400	4B12	8B12

**Table 4:** Mechanical properties of the concrete.

Grade of Concrete	Axial Compressive Strength/MPa	Modulus of Elasticity/MPa
C20	15.3	26,916
UHPC	109.3	42,900

**Table 5:** Mechanical properties of the rebar.

Grade of Reinforcement	Diameter/mm	Yield Strength/Mpa	Ultimate Strength/Mpa	Modulus of Elasticity/MPa
HRB335	12	393	542	2.08 × 10^5^

**Table 6:** The numerical bearing capacity of the specimens.

Specimen Number	Core Column Section Dimensions/mm	Section Dimensions of the Reinforced Rear Column/mm	Specimen Length/mm	Calculated Value/KN
ZY-2	200 × 200	300 × 300	2400	1722
JGZ-1	200 × 200	260 × 260	2400	1935.7
JGZ-2	200 × 200	300 × 300	2400	3307

## Data Availability

The raw data supporting the conclusions of this article will be made available by the authors on request.
